# Phospholipid Oxygen Microbubbles for Image-Guided Therapy

**DOI:** 10.7150/ntno.43808

**Published:** 2020-02-28

**Authors:** Traci D. Reusser, Kang-Ho Song, David Ramirez, Richard KP Benninger, Virginie Papadopoulou, Mark A. Borden

**Affiliations:** 1 Department of Mechanical Engineering, University of Colorado, Boulder, CO, USA.; 2 Department of Bioengineering, University of Colorado Anschutz Medical Campus, Aurora, CO, USA.; 3 Department of Biomedical Engineering, University of North Carolina at Chapel Hill and NC State, Chapel Hill, NC, USA.; 4 Biomedical Engineering Program, University of Colorado, Boulder, CO, USA.

**Keywords:** DBPC, size isolation, ultrasound contrast agent, tumor hypoxia, radiation therapy

## Abstract

In recent work, oxygen microbubbles (OMB) have been shown to oxygenate hypoxic tumors, increase radio-sensitivity and improve tumor control by radiation therapy. Compared to intra-tumoral injection, intravenous delivery of adjuvant agents such as OMBs for radiotherapy offers an attractive means of achieving true theranostic function in a minimally invasive manner via contrast-enhanced ultrasound (CEUS), while reducing the risk of injury, infection or displacing tumor cells. However, short intravascular circulation times with conventional DSPC-lipid OMBs may lead to premature off-target dissolution of OMBs with an associated reduction in tumoral oxygen delivery. Prior work on microbubble stability and gas exchange suggests that increasing phospholipid acyl-chain length of the encapsulating shell and OMB size may increase circulation persistence, delivery and dissolved oxygen content. In the following studies, we investigate the effect of two phospholipid shell compositions, DSPC (C18:0) and DBPC (C22:0), as well as three size distributions (0.5-2 µm, 2-10 µm and polydisperse) on OMB circulation persistence utilizing CEUS in the kidneys of live C57B1/6 male and female mice, six weeks of age. DBPC OMB formulations demonstrated increased circulation half-lives versus DSPC formulations (2.4 ± 1.0 *vs.* 0.6 ± 0.5 s, p<0.01 for 2-10 µm), as well as an increased maximum intensity by over tenfold (p<0.01). Size-dependent effects remained consistent across both formulations with larger 2-10 µm microbubbles demonstrating significantly increased half-lives (2.4 ± 1.0 *vs*. 0.3 ± 0.2 s, p < 0.01) compared to smaller 0.5-2 µm formulations of DBPC. These studies indicate that DBPC 2-10 µm OMBs may be improved adjuvant agents for radiotherapy with significant potential for CEUS interrogation.

## Introduction

It is estimated that approximately 40% of men and women in the U.S. will be diagnosed with cancer at some point in their lifetime 1. Tumor hypoxia remains a persistent challenge to improving the safety and efficacy of radiation therapy and chemotherapy. Oxygen microbubbles (OMB) are a minimally invasive solution for treating tumor hypoxia and have been shown to increase dissolved oxygen content *in vitro* 2. Furthermore, OMB can be imaged *in vivo* by ultrasound and triggered to release their oxygen cargo, thereby enabling image-guided therapy. In 2016, Owen et al. showed that orally administered OMB can reduce tumor hypoxia 3. In 2018, Fix et al. showed that intra-tumoral injection lipid-coated OMB can increase tumor oxygen levels and tumor control in a fibrosarcoma rat model 4. In the same year, Eisenbrey et al. showed that intravenous injection of surfactant-coated OMB can increase tumor oxygen and control in a breast cancer model 5,6. These studies have demonstrated the promise of ultrasound-guided OMB for treating hypoxic tumors.

**Figure 1:**
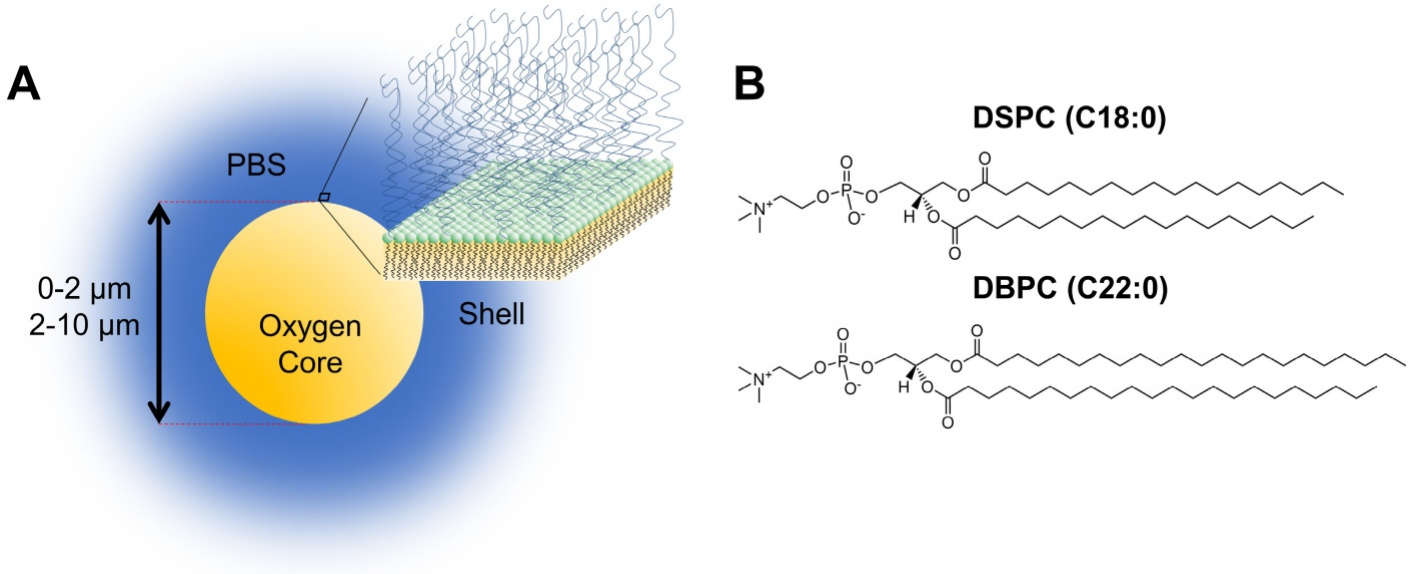
** A)** Cartoon illustration of OMB design for *in vivo* studies. The core of the microbubble is oxygen and is stabilized by a phospholipid monolayer shell comprising of **(B)** two acyl-chain phospholipids: DSPC and DBPC.

Despite these advances in oxygenation and control of hypoxic tumors, OMB used in these studies have relatively short circulation lifetimes and can limit the time window for radiotherapy. However, work with perfluorocarbon microbubbles has shown that *in vivo* lifetime can be greatly extended by inhibiting dissolution through choice of the phospholipid shell composition 7. The goal of this study was to redesign the phospholipid OMB to improve circulation lifetime for intravenous administration, in order to improve the clinical translatability of this promising theranostic technology (Fig. 1A). The dual nature of microbubbles for imaging and therapy provides a vehicle for ultrasound-mediated nanotheranostics 8-10.

Here, we report on a novel OMB comprising a long acyl-chain phospholipid designed for long circulation persistence following noninvasive intravenous administration. We examined the effect of shell composition, specifically acyl-chain phospholipids, on *in vivo* circulation persistence using DSPC (C18:0) and DBPC (C22:0) (Fig. 1B). Both size and composition were shown to affect *in vitro* and *in vivo* stability for perfluorocarbon microbubbles 7,11. The prevalence of phospholipid formulations (i.e. DEFINITY and SONOVUE) in contrast-enhanced sonography, as well as the tendency for lipids to be degraded readily once dissociated from the OMBs, suggests that the DBPC formulation should be safe for clinical translation. We also examined the effect of different microbubble sizes generated by differential centrifugation 12. Microbubble volume dose, MVD, was kept constant at 300 µL/kg throughout experiments 13,14. Equating MVD in 0.5-2 µm and 2-10 µm populations results in a much higher count and concentration of 0.5-2 µm, microbubbles due to their significantly smaller gas core volume. *In vivo* stability was quantified by the contrast persistence of OMB perfusing into the kidneys of C57B1/6 mice using a commercial small-animal ultrasound scanner. Despite significant differences in perfusion compared to a tumor, the kidneys were chosen as regions of interest due to their consistent physiology and morphology compared to a more typical xenograft tumor model. Indeed, tumors may exhibit a wide range of vascularization, which highlights the importance of examining OMB performance in a wider range of radiotherapy applications.

## Materials and Methods

### Materials

Phosphate buffered saline (PBS) solution was prepared by diluting 10× stock solution from Fisher Scientific International, Inc. (Hampton, NH, USA) 9:1 with deionized water (Direct-Q, Millapore, Billerica, MA, USA) and filtered through 0.2-µm diameter nylon filter attached to a vacuum. High purity oxygen was obtained from Airgas (Radnor, PA, USA). The two phospholipids, 1,2-distearoyl-sn-gylcero-3-phosphocholine (DSPC) and 1,2-dibehenoyl-sn-glycero-3-phosphocholine (DBPC) and the emulsifier, 1,2-distearoyl-sn-glycero-3-phosphoethanolamine-N-(polyethylene glycol 2000) (DSPE-PEG2000) were purchased from Avanti Polar Lipids (Alabaster, AL, USA).

### Microbubble Preparation

Individual lipid solutions were prepared by combining the different acyl-chain length phospholipids with DSPE-PEG2000 in a 9:1 molar ratio, respectively, to a final lipid concentration of 12 mg/mL. The lipids were dissolved in PBS solution, gently stirred using a magnetic stir bar and heated to reach their main phase transition temperature to make a homogeneous suspension. The lipid suspension was then sonicated using Branson Digital Sonifier SFX 550 (Danbury, CT, USA) for 10 min at 30% power to disperse lipids into small unilamellar liposomes 12. Lipid solutions were then cooled to 4°C before OMB generation.

OMBs were synthesized using an ultrasonic horn reactor enclosed in a water-cooled, continuous-flow chamber (Branson, Danbury, CT, USA) 15. Lipid solutions were flowed through the chamber with room temperature oxygen gas at 100% sonication power and then collected into a glass collection column with oxygen gas headspace. The final OMB microfoam was collected into 30-mL syringes (BD, Franklin Lakes, NJ, USA) to be further processed by centrifugation using a bucket-rotor centrifuge (Eppendorf 5804, Hauppauge, NY, USA). Microbubble cake was collected by centrifuging the initial OMB suspension at 130 relative centrifugal force (RCF) for 3 min. Excess lipid solution from the infranatant was collected and reused to generate more OMB. The final cake was collected into a 30-mL syringe to be further size selected by differential centrifugation 12. The cake was diluted to 30 mL with oxygen saturated PBS and centrifuged at 130 RCF for 2 min and washed three times to exclude OMB that were smaller than 2-µm diameter. The infranatant was collected and concentrated resulting in 0.5-2 µm OMBs. The cake was again diluted to 30-mL with oxygen saturated PBS and centrifuged at 30 RCF for 1 min to exclude OMB that were larger than 10-µm diameter. The infranatant was collected, containing OMB between 2-10 µm diameter. The infranatant was concentrated at 130 RCF for 4 min and was transferred to a 12-mL syringe (Covidien Monoject, Mansfield, MA, USA) and washed with PBS three more times with the final product being concentrated 2-10 µm diameter OMB. To reduce degradation in storage, OMBs were refrigerated and stored in gastight syringes—we note that the timescale of coalescence is on the order of several days, while the timescale of microbubble dissolution and clearance *in vivo* is on the order of seconds to minutes 7.

### Particle Sizing

Oxygen microbubbles were characterized by size, concentration and gas content. Microbubble size and concentration was measured using laser light obscuration and scattering (AccuSizer 780, NICOMP Particle Sizing Systems, Santa Barbara, CA). Gas volume fraction was measured by weight and volume of the OMB sample 16,17. Microbubble volume dose, i.e., oxygen gas volume injected per weight of animal, was calculated by using OMB concentration, size distribution and total injection volume 14. Microscopy was used as a visual inspection of microbubble formation and size using an Olympus BX52 (Center Valley, PA, USA) microscope at 40× magnification, and images were post processed using ImageJ (National Institutes of Health, Bethesda, MD).

### *In vivo* Imaging

All animal experiments were approved by the University of Colorado Denver Institutional Animal Care and Use Committee. Contrast persistence studies were performed in male and female C57B1/6 mice six weeks of age. Mice were first anesthetized using 2% isoflurane with oxygen carrier gas and placed supine on a heated platform. While the significance of isoflurane in regards to OMB longevity is somewhat unclear, it is important to note that anesthesia carrier gas has been indicated to adversely affect *perfluorocarbon* microbubble longevity 18,19. Heart rate, respiratory rate and temperature were monitored using a Vevo 2100 Physiological Monitoring Unit. Mice were kept under anesthesia via nose cone for the duration of the experiment. Hair was removed from the lower left kidney region using Nair hair removal lotion (Church & Dwight, USA). A modified 27-gauge, one half-inch winged infusion catheter (Terumo BCT, Lakewood, CO) was installed in the mouse tail vein for the duration of the sonography. The small diameter of the mouse tail vein dictated the use of the 27G needles, and manual injection was approximated to be 50 uL/s. While this flow rate has been demonstrated by previous studies to result in minimal to no microbubble destruction 20, it is important to note that the potential for microbubble destruction exists.

A VisualSonics Vevo 2100 small-animal ultrasound imaging scanner (Toronto, ON, Canada) with an MS250, 18-MHz transducer at 10% power was placed on the shaved kidney region using acoustic coupling gel (Medline Aquasonic, IL, USA). Mice were injected with OMB (MVD = 303.7 ± 9.8 µL/kg) while continuously imaging. Each mouse was imaged once per imaging session and then removed from anesthesia and placed in a recovery cage on top of a heating pad. Once conscious, the mouse was returned to its cage.

### Data Analysis

The Vevo 2100 small-animal ultrasound generated Digital Imaging and Communications in Medicine (DICOM) files that were used to extract individual video frames and real time measurements. These files were post-processed using the MATLAB R2018b DICOM reader (MathWorks, Natick, MA, USA) and were analyzed for gray scale intensity versus time. For *in vivo* mouse studies, B-mode was used to locate the kidney and Contrast Mode was used to measure the change in contrast intensity over time. The region of interest (ROI) was chosen as the entire kidney rather than just a portion of the kidney because signal attenuation and shadowing showed minimal effect. The average video intensity was determined over the entire ROI for each frame. The data was baseline adjusted and plotted as mean intensity versus time. Envelope detection in MATLAB was used to adjust for respiratory motion of the mouse and produce a smoothed time intensity curve (TIC). It is important to note that while half-life measurements should be independent of transducer-frequency-dependent factors, the maximum intensity and AUC results may be influenced by nonlinear sensitivity to microbubble size. Data was fit to a one-phase exponential decay model using GraphPad Prism version 8.3.0 for Windows (GraphPad Software, San Diego, CA, USA). The decay rate was used to determine the half-life of the microbubble. Total integrated enhancement (area-under-the-curve, AUC) was measured using the TIC data. All statistical analyses were performed using GraphPad Prism. Results are presented as mean ± standard deviation. For *in vivo* experiments, five to six mice were imaged for each lipid acyl-chain and size category. Statistical significance was evaluated using a Mann-Whitney comparison test.

## Results

### Polydisperse oxygen microbubbles

**Figure 2:**
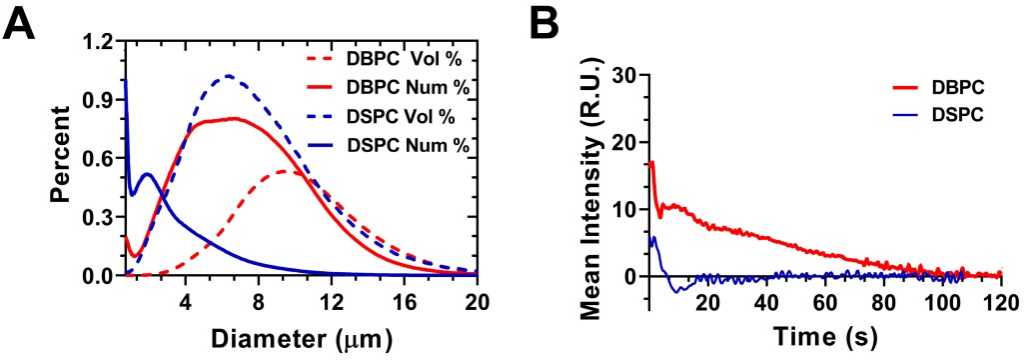
Interaction between polydisperse OMB size distribution and TIC. **A)** Accusizer number and volume weighted size distributions for polydisperse DSPC and DBPC. Sauter-mean and median diameter for DSPC (mean: 4.5 ± 0.9 µm; median: 4.1 ± 0.9 µm) and DBPC (mean: 5.9 ± 1.1 µm; median: 5.5 ± 1.4 µm). **B)** TIC curves for DSPC and DBPC.

Figure 2A shows the size distributions of polydisperse OMB for both lipid acyl-chain lengths (DSPC and DBPC). The number percent size distribution showed a peak ranging from 0.5-2 µm diameter and another peak ranging from 2-10 µm diameter. Volume percent showed a monomodal distribution for both lipid acyl-chain microbubble shell compositions. The volume percent had a size range from approximately 2-10 µm in diameter for both lipid acyl-chain microbubble shell compositions, with some OMB greater than 10-µm diameter. The initial OMB concentration was measured to be 6.0 × 10^9^ /mL for DSPC and 8.5 × 10^9^ /mL for DBPC. After concentration by centrifugation, the volume fraction was 71% for DSPC and 79% for DBPC. The OMB were diluted using oxygen-saturated PBS to a volume fraction of 50% for *in vivo* intravenous injections.

Mean video contrast enhancement and persistence in the blood stream was analyzed from the TICs (Fig. 2B). Injection of DBPC OMB showed a significant increase in contrast and persistence *in vivo*. The half-life of DBPC was 34.4 ± 16.4 s. Conversely, OMB comprising DSPC did not show a measurable contrast increase above noise. Some sparse contrast was observed at the time of injection, indicating that some OMB were present, but it could not be reliably quantified due to the low signal-to-noise ratio.

### Size-isolated oxygen microbubbles

**Figure 3:**
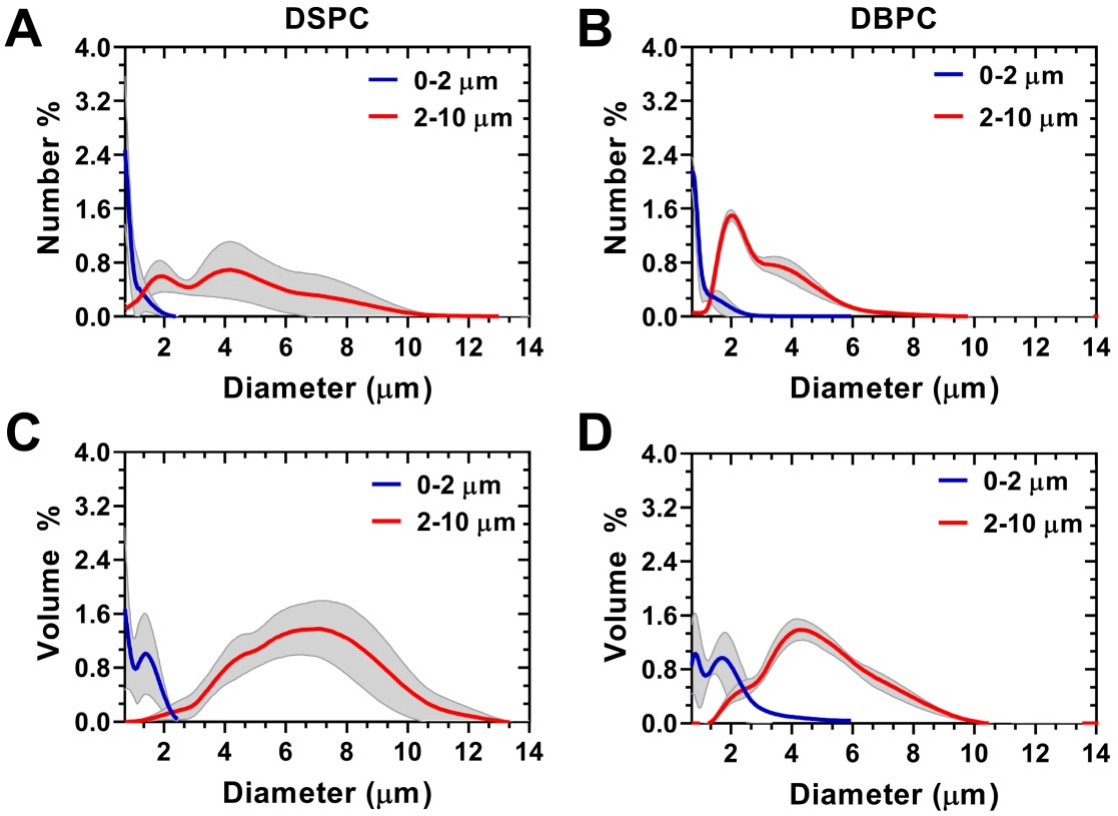
Accusizer size distributions with mean (solid lines) and standard deviation (gray shading, n = 3) for size-selected OMB. Number-weighted size distributions for: **A)** DSPC and **(B)** DBPC. Volume-weighted size distributions for: **C)** DSPC and **(D)** DBPC. Sauter-mean and median diameter for DSPC 0.5-2 µm (mean: 0.9 ± 0.1; median: 0.8 ± 0.1 µm), DSPC 2-10 µm (mean: 5.1 ± 1.0; median: 4.9 ± 1.1 µm), DBPC 0.5-2 µm (mean: 1.3 ± 0.1; median: 1.2 ± 0.1 µm), and DBPC 2-10 µm (mean: 6.1 ± 0.7; median: 5.7 ± 0.7 µm).

Size-selection was conducted to obtain OMB in two size categories, 0.5-2 µm and 2-10 µm diameters, for both DSPC and DBPC (Fig. 3). Concentration, total volume injected, total OMB count, and MVD for each phospholipid acyl-chain are shown in Table 1. After concentration by centrifugation, the volume fraction was 64% for DSPC and 73% for DBPC for the 0.5-2 µm size range and 53% for DSPC and 52% for DBPC for the 2-10 µm size range. The OMB were diluted using oxygen-saturated PBS to a constant volume fraction of 50% for *in vivo* animal injections.

**Figure 4:**
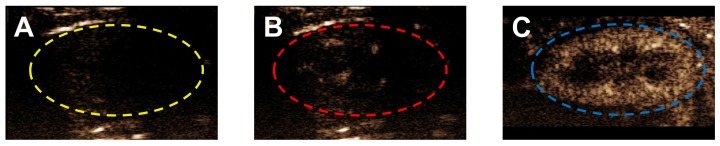
Ultrasound contrast enhancement in a typical mouse kidney following bolus injection of different lipid acyl-chain OMB, all sized to 2-10 µm, with MVD = 303.7 ± 9.8 µL/kg and volume fraction of 50%. **A)** Grayscale image of a mouse kidney in contrast mode with no OMB. Typically, a small ROI is used to avoid attenuation and shadowing effects; however, there were no indications of these effects and the entire kidney, indicated by a dashed line, was used for the ROI. Contrast enhancement in the kidney at peak enhancement after injection with OMB comprising **(B)** DSPC and **(C)** DBPC.

*In vivo* contrast persistence was measured using Vevo 2100 small-animal ultrasound with an 18-MHz transducer placed on the left mouse kidney. All mice were given bolus OMB injections. The MVD was kept constant at 303.7 ± 9.8 µL/kg. Figure 4 shows the ultrasound contrast signal enhancement following intravenous injection of OMB coated with either DSPC or DBPC.

**Figure 5:**
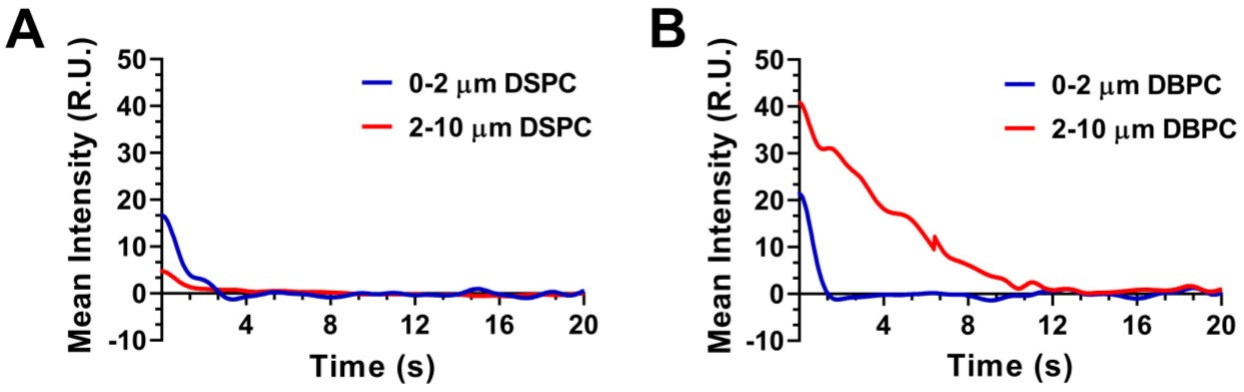
Effect of lipid acyl-chain and OMB size on *in vivo* ultrasound contrast persistence. Decay time intensity curve: **A)** DSPC and **(B)** DBPC.

Mean video contrast enhancement and persistence in the blood stream were analyzed from the TICs (Fig. 5). Injection of 2-10 µm DBPC OMB showed a significant increase in contrast and persistence compared to 2-10 µm DSPC OMB. Although 0.5-2 µm DBPC OMB showed an increase in contrast, the 0.5-2 µm OMB did not persist as long as the 2-10 µm OMB. In comparison, OMB comprised of DSPC, size 0.5-2 µm and 2-10 µm diameter, showed significantly less of an increase in contrast and persistence *in vivo*.

**Figure 6:**
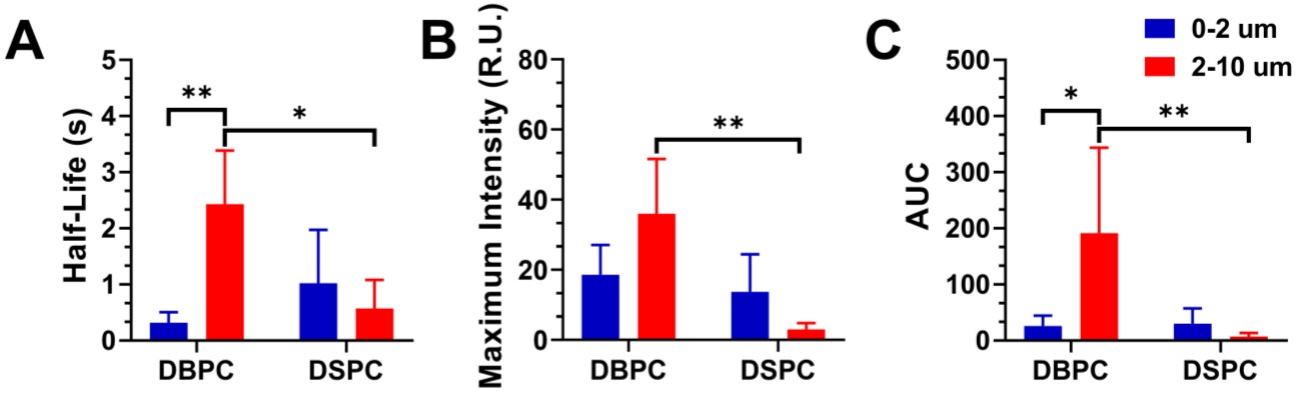
Effect of OMB formulation and size on pharmacokinetic parameters for contrast persistence *in vivo*. **A)** Mean half-lives of 0.5-2, 2-10 µm DBPC; and 0.5-2, 2-10 µm DSPC OMBs. **B)** Maximum ultrasound contrast intensity for each OMB formulation. **C)** AUC for each OMB formulation based on the pharmacokinetic model fit to the TIC. Statistical significance indicated for p < 0.05 (*) and p < 0.01 (**).

Figure 6 shows the resulting half-life, maximum intensity, and AUC values for the different OMB formulations. The half-life was determined by fitting the decay TIC to a one-compartment pharmacokinetic model. DBPC OMB formulations resulted in increased circulation half-lives versus DSPC formulations of all sizes (0.3 ± 0.2s vs 1.0 ± 0.9s, no statistical difference; 2.4 ± 1.0s vs 0.6 ± 0.5s, p < 0.01) for 0.5-2 µm and 2-10 µm, respectively. Size-dependent effects for DBPC with larger microbubbles demonstrated a significant increase in half-life (2.4 ± 1.0s vs 0.3 ± 0.2s, p < 0.01) compared to smaller microbubbles.

## Discussion

In this study, we demonstrate that OMBs comprising the long acyl-chain phospholipid, DBPC (C22:0), show an increase contrast enhancement and persistence *in vivo*. We also demonstrated that DBPC OMB between 2-10 µm diameter had the largest increase in contrast and persistence *in vivo* when compared to the smaller DBPC OMB between 0.5-2 µm diameter. The shorter acyl-chain phospholipid, DSPC (C18:0), showed minimal contrast enhancement and short persistence *in vivo*. This result is consistent with contrast agent studies that compared differing acyl-chain phospholipids for increase contrast persistence and circulation *in vitro* and *in vivo* 7. Longer acyl chains increase intermolecular forces between the phospholipids 21, thereby increasing shell rigidity 22,23, decreasing shell permeability 24,25, and increasing microbubble lifetime 7,26.

**Figure 7:**
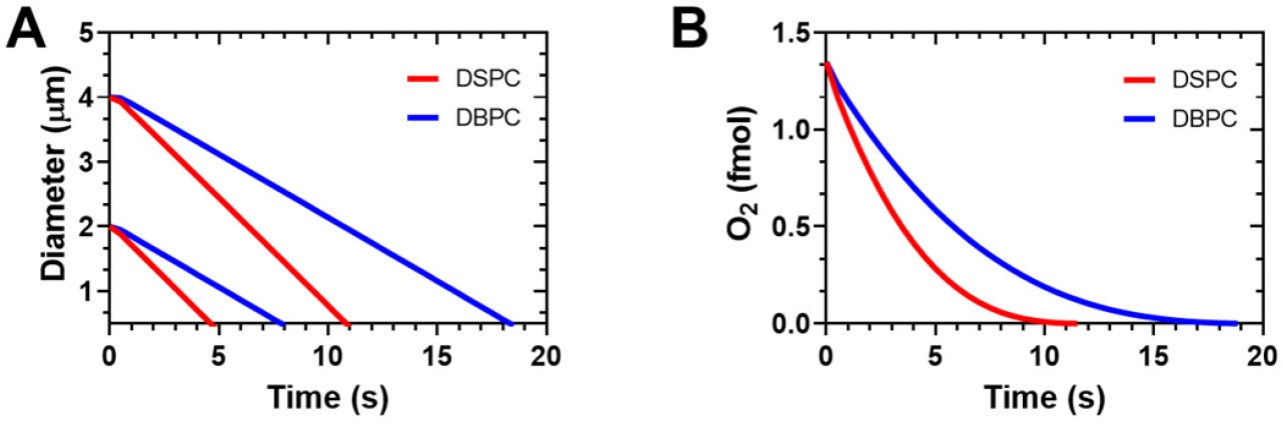
Simulation results on OMB gas exchange and dissolution in arterial blood with an inspired oxygen fraction of 1.0 (oxygen carrier). A) Diameter vs. time curves for DSPC and DBPC for initial diameters of 2 and 4 µm. B) Encapsulation oxygen content in 4-µm diameter OMBs vs. time. Simulations were carried out using the multi-gas exchange model and lipid parameters determined by Kwan and Borden 27,28. Arterial blood gases were PaO2 = 546 mmHg and PaCO2 = 61 mmHg determined experimentally from Mullen et al. 18.

This data corresponds well with model simulations of microbubble gas exchange and dissolution based on the model developed by Kwan et al. 27,28. The microbubbles were simulated in an arterial blood environment when the inspired oxygen fraction is 1.0 18, as in our experiments. The model accounts for differences in lipid mechanics during growth and dissolution to the initial diameter during gas exchange and for the lipid permeation resistance during the entire process. The model neglects lipid mechanics during dissolution below the initial diameter. Figure 7A shows the expected diameter-time curves for microbubbles coated with the two different lipid species. DBPC provides superior stability owing to its higher gas permeation resistance. Additionally, the larger microbubbles take longer to dissolve. These two effects combined provide a robust stability in the arterial gas environment. The greater stability of the DBPC microbubbles also leads to longer duration of oxygen retention in the microbubble (Fig. 7B). The simulation results agree with the experimental results shown above both qualitatively with respect to the trends and quantitatively with respect to dissolution time. This result is somewhat surprising given the absence of lipid mechanical effects for the dissolution phase of the simulation, and the simplified mass transfer assumptions.

It is interesting that the injections of microbubble populations follow the general trend for the simulation results of individual microbubbles. While the smaller OMBs are expected to dissolve more quickly than the larger ones, they are also much more numerous and contain overall the same microbubble volume dose (Table 1). We have seen in prior experiments with perfluorobutane gas that, when matching to MVD, different sized microbubbles have essentially the same half-life 11. Matching MVD also gave similar results for different sized microbubbles in blood-brain barrier disruption studies 13. It is curious then, that even though the OMBs were matched to MVD here, we observe longer circulation persistence for larger OMBs. Perhaps this is due to the different encapsulated gases: whereas perfluorocarbon may rapidly saturate the blood and tissue compartments, oxygen gas may not saturate owing to metabolism and binding to proteins and enzymes, such as hemoglobin. This highlights the importance of future studies to measure the pharmacokinetics and biodistribution of microbubbles containing different gases and encapsulated with different shells. Indeed, the removal of microbubbles in circulation can primarily be attributable to certain organs, such as the liver, spleen, kidneys and lungs. Injection site may therefore affect microbubble pharmacokinetics, and furthermore injection into larger vessels, wider bore needles or slower injection speeds may reduce shear stress and destruction of oxygen microbubbles to enhance delivery to the tumors.

On the topic of oxygen delivery for radiotherapy, Fix et al. has previously shown that a 30% increase in tumoral hemoglobin saturation is sufficient to achieve a ~30% reduction in tumor growth rate by injection of ~3×10^10^ OMBs/kg directly into the tumor 29. For intravenous, as opposed to intratumoral administration, we see a constraint on OMB concentration (<5×10^8^ OMB/mL) due to handling considerations related to excessive viscosity at high OMB concentrations. While there are several complicating parameters in a practical comparison of intratumoral and intravenous delivery, we suspect that the necessary intratumoral oxygen level may be achieved with serial or continuous intravenous application of the significantly more persistent DBPC OMBs. Effect of microbubble size and composition on circulation persistence and oxygen payload delivery is expected to occur for continuous infusion, as well as the bolus injection shown in the present work.

It is important to note that OMBs exhibit reduced lifetimes compared to perfluorocarbon microbubbles (2-3 seconds for DBPC OMB vs. 30 min for DBPC perfluorocarbon MBs) due to the greater solubility of oxygen compared to perfluorocarbon gases in water 7. While the DBPC formulation significantly increased the longevity of OMBs under ultrasound compared to DSPC and smaller microbubble formulations, this limitation in longevity may still necessitate a timely intravenous or intratumoral injection to maximize efficiency in regards to radiotherapy. Indeed, we suspect the use of longer-lived OMBs is an important aspect of preserving and localizing the oxygen to a tumor, and will be integral to any theranostic OMB application.

Finally, future work will likely involve further improving microbubble longevity, which may be achieved by 1) partially replacing some of the oxygen gas-core with hydrophobic gas, 2) further reducing the immunogenicity of the microbubble utilizing external moieties (i.e., CD47) and 3) increasing the microbubble volume dose (MVD) as previously demonstrated with perfluorocarbon lipid microbubbles 11,13.

## Conclusion

In this study, we demonstrate increased circulation *in vivo* with novel DBPC OMBs. Oxygen microbubbles were designed with two acyl-chain phospholipid shells, DSPC and DBPC, to improve MB stability for oxygen delivery to hypoxic tumors. Results showed that the existence of a bimodal TIC resulting from a polydisperse size distribution supports the size dependent effect on OMB contrast and circulation persistence. Larger diameter 2-10 µm OMBs have increased circulation persistence and contrast *in vivo* for both lipid acyl-chain lengths. These studies indicate that, when compared to previous OMB formulations, 2-10 µm DBPC OMBs have a significantly increased contrast persistence, contrast intensity, and improved circulation lifetime, making them ideal theranostic agents for radiotherapy, with significant potential for CEUS integration.

## Figures and Tables

**Table 1:** OMB mean parameters and standard deviation for *in vivo* studies

	DBPC (C22:0)		DSPC (C18:0)	
	0.5-2 µm	2-10 µm	0.5-2 µm	2-10 µm
Injected volume [µL]	65 ± 28	43 ± 4	128 ± 73	55 ± 5
OMB concentration [× 10^9^ /mL]	2.3 - 3.5	1.4 - 19.0	19.8 - 112.0	0.04 - 0.9
Total OMB injected [× 10^9^]	0.3 ± 0.1	0.5 ± 0.6	12.6 ± 8.7	0.8 ± 1.3
MVD [µL/kg]	307.1 ± 16.1	296.0 ± 5.4	303.0 ± 10.1	300.3 ± 8.4
